# Influence of Parental and Offspring Dietary Behaviors on the Association of Overweight and Obesity between Two Generations: Results from a Cross-Sectional Analysis of Parent-Offspring Trios in China

**DOI:** 10.3390/nu14214625

**Published:** 2022-11-02

**Authors:** Qi Ma, Ting Chen, Jieyu Liu, Manman Chen, Di Gao, Yanhui Li, Tao Ma, Xinxin Wang, Li Chen, Ying Ma, Yi Zhang, Yanhui Dong, Yi Xing, Jun Ma

**Affiliations:** 1 National Health Commission Key Laboratory of Reproductive Health, Institute of Child and Adolescent Health, School of Public Health, Peking University, Beijing 100191, China; 2 Key Laboratory of Environmental Factors and Chronic Disease Control, School of Public Health and Management, Ningxia Medical University, No. 1160, Shengli Street, Xingqing District, Yinchuan 750004, China

**Keywords:** dietary behaviors, overweight/obesity, parents, offspring, cross-sectional study

## Abstract

Limited evidence exists on the influence of dietary behaviors on the association of overweight/obesity between parents and offspring. This study aimed to investigate the influence of dietary behaviors on the association of overweight/obesity between two generations, and puts forward strategies for preventing childhood obesity. Data were obtained from a cross-sectional survey conducted in China; a total of 40,197 parent-offspring pairs were included. Overweight/obesity was defined based on the body mass index and waist circumstance; the association of overweight/obesity between two generations was evaluated by multivariate and binary logistic regression and stratified analyses. Compared with the offspring who were free of parental overweight/obesity, the ORs of offspring with both parental overweight/obesity reached 2.66, 1.72 and 4.04 for general, simple abdominal and compound obesity. The offset effect of dietary behaviors was observed on the association between parental obesity and the offspring’s general or simple abdominal obesity, with non-significant ORs when parents or/and offspring had healthy dietary behaviors. It was difficult for a healthy diet alone to offset the high heritability and intergenerational transmission of childhood obesity caused by parental obesity. Multifaceted improvement of lifestyle behaviors, and a combination of individual and family engagement, could be targeted measures to control childhood obesity.

## 1. Introduction

Childhood overweight and obesity (OW/OB) has become one of the most serious public health challenges of the 21st century. The number of children and adolescents affected by OW/OB has increased more than ten times, from 11 million in 1975 to 124 million in 2016 [1], and an estimated alarming level of 40 million children will suffer from OW/OB in the next decade [2]. Children and adolescents with OW/OB experience difficulties in breathing, high risks of fractures, hypertension, early markers of cardiovascular disease, insulin resistance, and psychological effects [3,4], and they might be also at high risk of premature death and disability in adulthood [5,6].

The resemblance of offspring and their parents’ weight status has been the focus of long-term interest, with researches suggested there is an intergenerational transmission of OW/OB from parents to their offspring [7,8]. Besides the genetic component confirmed in the twin and adoption study [9], parents have always had a strong impact on their child’s lifestyle behaviors [10,11], which could contribute to offspring OW/OB. In addition, parental OW/OB might be a risk factor for cardiovascular diseases [12], asthma [13], and metabolic syndrome [14] in their offspring. Although the conclusions were consistent that parental OW/OB was associated with offspring body mass index (BMI) levels, few studies investigated the potential modified effects of other factors on such associations.

Notably, it is widely acknowledged that unhealthy dietary behaviors contribute to weight status change [15]. For example, excess intake of sugar-sweetened beverages (SSBs), high energetic foods, fried snacks and meat, as well as breakfast skipping were associated with OW/OB in children and adults [16,17]. Studies from the UK [18] and Ireland [19] found that parental dietary behaviors influenced the intergenerational transmission of overweight and obesity, but the evidence about whether dietary behaviors may regulate the association of OW/OB between parents and offspring is insufficient in China to date.

To fill these knowledge gaps, based on a large and national representative data, we aimed to validate the association of parental–offspring OW/OB, and examine the potential influence of parental or offspring dietary behaviors on the association between parental and offspring OW/OB, and therefore put forward strategies for the prevention of childhood OW/OB in China.

## 2. Materials and Methods

### 2.1. Study Design and Participants

**Figure 1:**
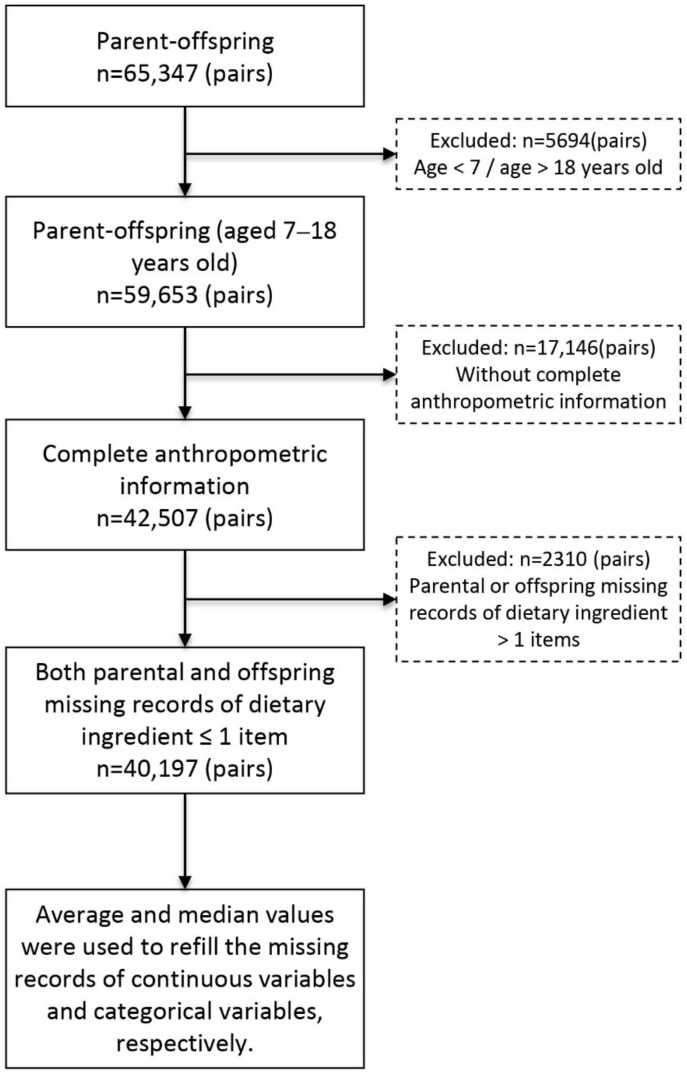
The selection process of the participants.

This is a re-analysis from a previous database, which was collected from a national cross-sectional study in China from seven provinces in 2013 [20]. More detailed information about this study was presented elsewhere [20]. Briefly, we used a multistage cluster random sampling to choose participants in national seven provinces. Several regions were randomly included in every province at the beginning. Then, 12–16 schools were randomly selected in each region. Two classes in every grade from each school were subsequently chosen at random, and their parents were also invited to engage in this survey. A total of 65,347 children and adolescents were included in the study. We chose 59,653 of the children aged between 7–18 years old, which is an age range with a high waist circumference screening threshold among children and adolescents in China. Anthropometric information was taken from 42,507 parent–offspring pairs. After removing participants who had more than one missing item from the group of dietary behaviors, a total of 40,197 children and adolescents remained in the final analysis, together with their parents (Figure 1).

All survey sites used the same protocol during the implementation process, and all processes of randomization were performed by a staff member who was not involved in the survey. This study was approved by the Medical Ethical Committee of Peking University (IRB No. 00001052-12072). All included students and their parents signed the written informed consent.

### 2.2. Data Collection

The childhood questionnaire was performed to collect information on sex, age, residential area, amount of moderate to vigorous physical activity (MVPA), and dietary behaviors. The parental questionnaire was distributed to collect information including single-child status, breastfeeding of offspring, self-reported height, weight, parental highest education attainment, MVPA, and the dietary behaviors of parents.

The self-administered child questionnaires were delivered to students (except grades 1–3) in a class meeting by the trained school teacher. Both parent and child questionnaires of children in grades 1–3 were filled by the parents. Trained project members interpreted all of the questionnaires in detail. Appropriate help and guidance were given by these project members as effectively as possible. The questionnaires would be rechecked by 3% within one week for the same participants.

All the information about the covariates in the questionnaire are presented in Supplementary Table S1. Parental educational attainment was grouped into “primary school or below”, “junior high school and senior high school” and “junior college or above”. In addition, the daily time of MVPA was calculated as daily time = (days of MVPA per week × time in those days)/7. According to the Physical Activity Guidelines for the Chinese Population (2021) [21], we defined physical inactivity as MVPA < 1 h/day in offspring and MVPA < 2.5 h/week in parents.

### 2.3. Anthropometric Measurement and Definition

Anthropometric measurements were conducted to measure the height, weight, and waist circumference (WC) of the children by trained investigators in schools, according to the basis of the Chinese Students’ Physical Fitness and Health Survey Report 2010 [22]. Children were asked to stand straight in light clothing and without shoes. Height was measured using the portable stadiometer with 0.1 cm precision, weight was measured to the nearest 0.1 kg by a Lever-type might scale, and WC was measured with an accuracy of 0.1 cm using a non-elastic tape at the end of a natural breath, at the midpoint between the top of the iliac crest and the lower margin of the last palpable rib. Every indicator was measured twice, and the average level of the two measurements was calculated for the final analyses. BMI was calculated as the weight (kg) divided by the square of the height (m^2^).

Conforming to sex-age-specific BMI cutoffs recommended by national standards [23] (Supplementary Table S2), we defined high BMI levels in offspring as BMI ≥ the “overweight” threshold of the corresponding sex and age group. We also defined the high WC levels as WC higher than the 90th percentile for sex and age [24] (Supplementary Table S3). Multiple obesity status was defined in our study including general obesity, simple abdominal obesity, compound obesity, and high WC and BMI levels. General obesity referred to high BMI levels but not high WC levels; simple abdominal obesity referred to lower BMI levels but high WC levels; compound obesity referred to both high BMI and WC levels [25] (Supplementary Table S4).

Parents were asked to report their height (cm) and weight (kg), while body mass index (BMI) was calculated as the weight (kg) divided by the square of the height (m^2^). Parental OW/OB was defined as BMI ≥ 24 kg/m^2^, according to the criteria established by the Working Group on Obesity in China [26]. In this study, we divided the study population according to the parental OW/OB status.

### 2.4. Definition of Healthy Dietary Behaviors

For both offspring and parental dietary behaviors, the consumption of SSBs, meat, frequency of intake of fast foods, and habits of having breakfast were included. Because the parental questionnaire was filled out by either parent, the dietary behaviors of one parent were used to represent the diet of both parents.

The frequency (days) and amount (serving per day) of dietary behaviors, including the total consumption of SSBs and meat over the past 7 days, were investigated. Students and their parents were asked “How many days, over the past 7 days, have you eaten meat or drunk SSBs? How many servings in one day?” [27,28], respectively. As set in the questionnaire, SSBs included common soda, orange juice and sugary juices; meat included all kinds of meat and meat products. One serving of SSB was determined as a canned beverage (approximately 250 mL), while one portion of meat equaled the size of an adult’s palm (approximately 100 g) [29]. Based on the information, participants answered the frequencies and quantities they ate. The weekly dietary intake was calculated as the number of intake days in a week multiplied by the number of servings per day. Participants were also asked “How many days, over the past 7 days, have you eaten breakfast?” and “How many days, over the past 30 days, have you eaten fast food?”. As set in the questionnaire, fast foods are referred to as fried chicken, hamburgers, French fries, and other kinds of foods sold in fast food restaurants. According to the information, participants answered the frequencies they ate.

According to The Chinese Dietary Guideline (2016) [30], The American Heart Association [31], and other previous studies [32], healthy dietary behaviors were defined as meeting three to four healthy dietary factors as (1) Drink SSBs ≤ 3 servings per week; (2) Eat meat ≤ 3 servings per week; (3) Eat breakfast ≥ 6 days per week; (4) Eat fast food < 1 day per month.

### 2.5. Statistical Analysis

Chi-square (χ^2^) tests and analysis of variance were used to examine the difference in categorical variables and continuous variables among groups, respectively; the Bonferroni correction was performed to make a multiple comparison. A multiple logistic regression model was adopted to calculate the odds ratio (OR) and 95% confidence intervals (95% CI), and to evaluate the association between multiple types of obesity for parents and offspring. We adjusted for some confounders including offspring age, sex, resident area, single-child status, breastfeeding status and MVPA status, as well as parental highest educational attainment and MVPA status. Binary logistic regression was also used to evaluate the association between parental OW/OB and the risk of high WC and high BMI levels in offspring, adjusting the same covariates.

To assess the influence of dietary behaviors on the association between parental OW/OB and offspring obesity, stratified analyses were conducted according to parental and offspring dietary behaviors. Moreover, a combined effect of offspring and parental dietary behaviors on the relationship of OW/OB between two generations was further explored. Spearman correlation coefficients were calculated to examine correlations between parental and offspring dietary behaviors.

We used the average value to refill the missing data of continuous variables via the option of “Series mean” in the section of SPSS named “Replace missing values”, while using median values of categorical variables to refill the missing records manually. To verify the correctness of the method that refills the missing data, a sensitivity analysis was performed.

All analyses were performed using IBM SPSS Statistics software (version 25.0, SPSS, IBM, Armonk, NY, USA). A two-sided *p*-value < 0.05 was considered statistically significant.

## 3. Results

### 3.1. Baseline Characteristics of the Study Population

Table 1 and Supplementary Table S5 show the characteristics of 40,197 parent-offspring pairs included in our study. The offspring were 10.9 (SD = 3.0) years old on average, and 52.7% (n = 21,197) of them were boys. Among them, 4034 (10.09%) offspring only had an OW/OB mother, 14,366 (35.93%) offspring only had an OW/OB father, and there were 5182 (12.90%) offspring whose parents were both OW/OB. The mean BMI was 24.1 kg/m^2^ (SD = 3.2) and 22.2 kg/m^2^ (SD = 3.0) for the participants’ fathers and mothers, respectively.

### 3.2. Association of OW/OB between Two Generations

We explored the association between parental OW/OB and offspring in three types of obesity, and high WC and BMI levels. Notably, the association between parental OW/OB and offspring obesity status was statistically significant when at least one parent was OW/OB, but the magnitudes of association reached the largest when both of their parents were OW/OB. Compared with the offspring who were free of parental OW/OB, the ORs(95% CI) of offspring with both parental OW/OB reached 2.66 (1.91–3.71) for general obesity, 1.72 (1.40–2.12) for simple abdominal obesity, and 4.04 (3.75–4.35) for compound obesity. Similar significant associations were observed for high WC (3.65 (3.38–3.93)) and high BMI (3.92 (3.65–4.21)) levels of offspring among those whose parents were both OW/OB (Table 2).

### 3.3. The Influence of Parental Dietary Behaviors on the Association of OW/OB between Two Generations

**Figure 2:**
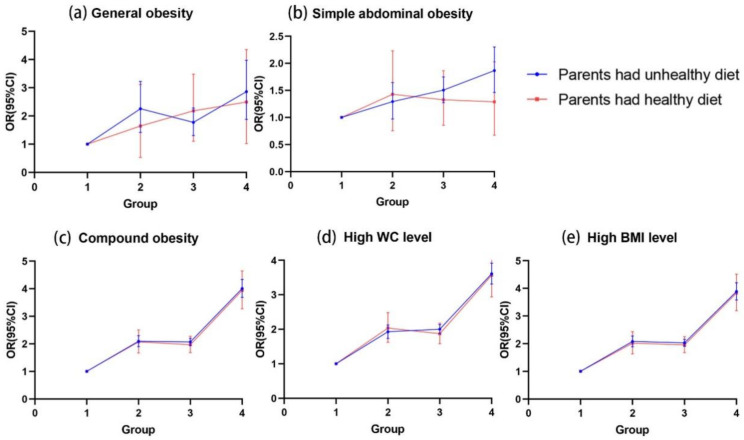
Association of OW/OB between parents and offspring, stratified by parental dietary behaviors. Notes: Adjust for offspring age, sex, resident area, single-child status, breastfeeding, SSBs consumption, meat consumption, breakfast eating, fast food consumption, and parental highest educational attainment. Group 1, offspring with no parental OW/OB; Group 2, offspring with only maternal OW/OB; Group 3, offspring with only paternal OW/OB; Group 4, offspring with both parental OW/OB.

We then assessed the relationship of OW/OB between two generations divided by parental dietary behaviors. Similarly, the results showed that parental OW/OB was associated with an increased risk of offspring’s multiple obesity, whether parents had healthy dietary behaviors or not. When parents had unhealthy dietary habits, the ORs (95% CI) of general obesity was 2.73 (1.88–3.79) in offspring with both parental OW/OB, higher than 1.73 (1.31–2.28) and 2.14 (1.42–3.22) in offspring with only paternal or maternal OW/OB. The same results could be observed in simple abdominal obesity, compound obesity, a high WC level, and a high BMI level. Among offspring whose parents had healthy dietary behaviors, parental OW/OB was associated with a higher risk of all types of obesity in the offspring. The exception was for simple abdominal obesity, in which ORs (95% CI) were non-significant (1.17 (0.67–2.03), 1.26 (0.85–1.86) and 1.30 (0.76–2.23) in offspring with both parental OW/OB, only paternal OW/OB, and only maternal OW/OB, respectively) (Figure 2, Supplementary Table S6).

### 3.4. The Influence of Offspring Dietary Behaviors on the Association of OW/OB between Two Generations

**Figure 3:**
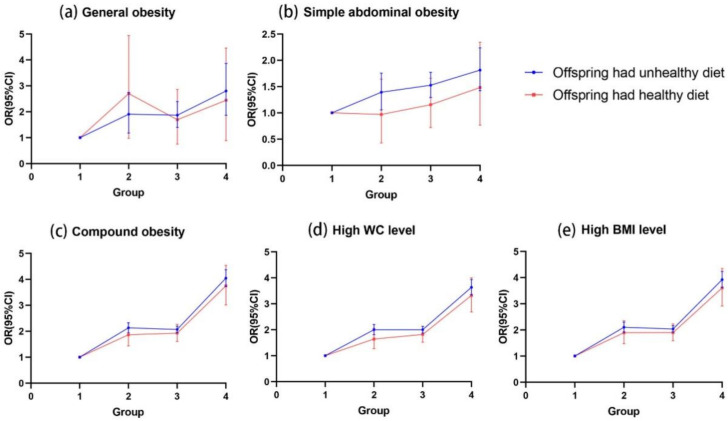
Association of OW/OB between parents and offspring, stratified by their offspring’s dietary behaviors. Notes: Adjust for offspring age, sex, resident area, single-child status, breastfeeding, parental highest educational attainment, parental SSBs consumption, meat consumption, breakfast eating, and fast food consumption. Group 1, offspring with no parental OW/OB; Group 2, offspring with only maternal OW/OB; Group 3, offspring with only paternal OW/OB; Group 4, offspring with both parental OW/OB.

We also analyzed the association between parental OW/OB and their offspring’s weight status, stratified by their offspring’s dietary behaviors. The results also confirmed whether or not the offspring had a healthy diet, and that parental OW/OB increased the risk of compound obesity, high WC levels, and high BMI levels in their offspring. The ORs (95% CI) of compound obesity among offspring adhering to healthy dietary behaviors were 4.03 (3.73–4.37) when both parents were OW/OB, 2.07 (1.94–2.20) when only the father was OW/OB, and 2.13 (1.94–2.33) when only the mother was OW/OB. Although the association between parental OW/OB with the offspring’s general and simple abdominal obesity has a similar trend when the offspring had unhealthy dietary behaviors, it did not reach significance when the offspring had healthy dietary behaviors (Figure 3, Supplementary Table S7).

### 3.5. Combined Effects of Parental and Offspring Dietary Behaviors on the Association of OW/OB between Two Generations

We first calculated the Spearman correlation coefficients to examine correlations between parental and offspring dietary behaviors. Parental dietary behaviors were weakly correlated with corresponding offspring dietary factors (Supplementary Figure S1). However, there was a stronger association between meat and fast food consumption between offspring and their parents, with the Spearman correlation coefficients of 0.28 and 0.32, respectively.

Further, we evaluated the combined effects of parental and offspring dietary behaviors on the association of OW/OB between two generations. The risk of the offspring’s general and simple abdominal obesity due to their parents’ OW/OB were almost non-significant, except both two generations had unhealthy dietary behaviors, whose ORs (95% CI) were 2.80 (1.87–4.18), 1.82 (1.35–2.44) and 1.98 (1.25–3.12) of general obesity when both parental, only paternal and only maternal were OW/OB. In contrast, parental OW/OB was always associated with the offspring’s compound obesity, high WC level, and high BMI level, even though both two generations had healthy dietary behaviors, and the risks were still higher in offspring with both parental OW/OB than offspring with only paternal or maternal OW/OB. When both parents and the offspring had unhealthy dietary behaviors, the ORs (95% CI) of compound obesity in offspring with both parental OW/OB was 4.05 (3.72–4.41), and only paternal OW/OB was 2.09 (1.96–2.23), only maternal OW/OB was 2.12 (1.92–2.35) (Table 3).

### 3.6. Sensitivity Analysis

The range of the number of each missing variable was 33–7358, less than 20% of the total number of participants (Supplementary Table S8). The results of the sensitivity analysis were similar to the main results, and did not change essentially when we restricted the study sample to participants without missing covariates (Supplementary Tables S9–S12).

## 4. Discussion

To our knowledge, parental OW/OB was strongly associated with multiple types of obesity status in offspring. Compared with offspring with only paternal or maternal OW/OB, the risk of obesity increased among offspring whose parents were both OW/OB. Different from our assumption, we found that parental and offspring healthy dietary behaviors had a slight influence on the association of OW/OB between two generations. Comprehensive strategies, including advocating healthy dietary behaviors, should be implemented when preventing OW/OB in children and adolescents.

Parental OW/OB was strongly associated with childhood OW/OB. Consistent with the present findings, studies from Ukraine and Iran found that children had higher BMI when their parents also had OW/OB [33,34]. The results of the WC-based analyses did not differ substantially from the BMI-based analyses [7]. In addition, we found that the association between parental OW/OB with offspring OW/OB was superimposed, because the risk of offspring OW/OB was highest when both parents were OW/OB. A cohort study found that both parents’ long-term overweight was the strongest single predictor for overweight in children [8], which also confirmed our finding. This may be partially explained by the increased genetic susceptibility to obesity due to genetically obesity-prone parents. Therefore, this study and several previous studies simultaneously confirmed that the prevention and control of obesity in offspring under the background of the obesity epidemic required not only actions from children themselves, but also parental participation.

A cohort study found the maternal dietary pattern during pregnancy was positively associated with offspring’s OW/OB when they were 5 years old, but no significant associations were observed for the paternal dietary patterns [19]. The reason for the inconsistent associations may be owing to the non-controlled breastfeeding covariable that is associated with the offspring’s OW/OB [35]. Meanwhile, the randomized controlled trial of the dietary behavior intervention for obese mothers found that the diet after pregnancy would not influence the offspring’s weight status [18]. In the present study, we used the dietary behaviors of one parent to represent the diet behaviors of both parents, and found parental or offspring dietary behaviors had slight influences on the association of OW/OB between two generations, with a significant association also observed even if both parents and offspring had healthy dietary behaviors. Corresponding to our findings, studies of twins [36] had suggested a strong genetic underpinning for obesity that can also be influenced by the family environment, but the effects were small [37]. Our findings need to be explained with caution, because we could not estimate the difference in parental dietary behaviors between perinatal and the current stage at which we collected the data.

The interaction between genetic and environmental factors is the underlying mechanism of the influence of dietary behaviors on the association of OW/OB between the two generations. Dietary behaviors are an important environmental factor. Previous reports concluded that excess intake of SSBs, fried snacks and meat, as well as breakfast skipping were associated with OW/OB in children and adults [16,17]. In addition, dietary nutrients are key factors in the mechanism [38]. A lower dietary saturated fat and energy intake at 28 weeks gestation-induced was associated with a reduction in infant subscapular skinfold thickness at 6 months of age [18]. If mothers adopted an unhealthy dietary behavior before or during the perinatal period, it may influence the process of DNA methylation of obese genes in the hypothalamus and placenta [39,40]. In addition, epigenetic inheritance owing to obesity and diet could be found on the paternal side. This epigenetic inheritance was stable and could not be changed by environmental factors [41]. For example, an adoption study from Denmark found there was a clear relationship between the adoptee’s weight and BMI of their biologic parents, but not the BMI of adoptive parents [42]. These evidences may support our finding that parental dietary behaviors are weakly correlated with the offspring’s dietary behaviors, but the association of OW/OB between them were prominent. The obvious modified effects of dietary behaviors were observed in general obesity and simple abdominal obesity in our study, which could explain that these two types of obesity were influenced by environmental factors more than genetic factors. However, we speculated that compound obesity, high WC (did not control the BMI), and high BMI (did not control WC) may mainly be influenced by genetic factors. There was a superimposed hereditary effect of BMI and WC, because 60% of the heritability of WC was common to BMI and 40% was due to different genetic factors [37], leading to a stronger effect of the gene.

This is the first study to explore the potential influence of dietary behaviors on the association of OW/OB between two generations in China. We provided a new explanation and enriched the connotation of the modified effects of well-known dietary factors on OW/OB in children and adolescents. However, such findings do not suggest that a healthy dietary behavior for controlling for OW/OB is useless when children might have a congenital risk for OW/OB. For example, strongly genetic conditions—notably, phenylketonuria—have proved to be entirely treatable by dietary behaviors interventions, a phenylalanine-free diet in the case of phenylketonuria. Thus, obesity intervention should not only focus on healthy dietary habits to eliminate the risks of obesity in offspring with OW/OB parents, other healthy lifestyles such as more physical activities and less sedentary time also should be considered. Longer-term weight control will require a combination of individual and family engagement and society-wide efforts to modify the environment, especially for children at high genetic risk.

The strengths of our study included a large sample size collected from seven provinces in China, including the eastern region with a developed economy and the western region with an underdeveloped economy, which made our findings generalizable to all populations in China. Since BMI has high specificity but low sensitivity to detect excess adiposity, and fails to identify over a quarter of children with excess body fat percentage [43], we used both BMI and WC to estimate the OW/OB in offspring, which had a higher specificity and sensitivity [44]. Based on the BMI and WC, we classified three types of obesity in offspring and included the two components separately. Previous studies showed that BMI and WC were good predictors of body fat percentage, and correlated with the incidence of cardiovascular disease in children and adolescents [45,46]. Thus, the present study could therefore present the association between parental OW/OB and the potential risks of cardiovascular disease in children and adolescents to some extent, due to the comprehensive assessment of overweight status in children.

Several limitations should be paid attention to. First, our study was a cross-sectional study which limited us to making casual inferences; therefore, further longitudinal studies were needed to confirm our findings. Second, the self-reported parental height and weight might not be as accurate as measured directly. However, self-reported height and weight in adults were found to be highly reliable in other studies [47,48]. Third, we did not collect the WC of parents so that we could not classify the parental obesity into different types. In addition, we concentrated on the dietary behaviors those are modifiable and have values of prevention, and collected the dietary information from a broad category, so we cannot calculate the quantities of dietary nutrients which are important to understand the mechanism of our findings. Lastly, our study only examined the influence of dietary behaviors on the association of overweight and obesity between two generations; other lifestyles in parents and offspring were not investigated. Prospective researches examining the potential offset effects of dietary nutrients and other lifestyles for such associations are needed.

## 5. Conclusions

Parental OW/OB was strongly associated with multiple types of obesity in offspring. Compared with offspring with only paternal or maternal OW/OB, the risk of obesity reached the highest among offspring whose parents were both OW/OB. Parental and offspring healthy dietary behaviors had a slight influence on the association of OW/OB between two generations. Thus, healthy diet recommendations alone in both parents and offspring might not be able to offset the high heritability and intergenerational transmission of childhood obesity caused by parental obesity. Longer-term weight control will require a multifaceted improvement of lifestyle behaviors, and a combination of individual and family engagement and society-wide efforts to modify the environment, especially for children at high genetic risk.

## Figures and Tables

**Table 1:** Descriptive characteristics of the study population stratified by parental OW/OB status.

		Parental OW/OB Status				
Characteristics	Total n = 40,197	Both Parental OW/OB n = 5182	Only Paternal OW/OB n = 14,366	Only Maternal OW/OB n = 4034	None OW/OB n = 16,425	*p*-Value
***Offspring factors***						
Age, year	10.9 ± 3.0	11.0 ± 3.0	10.7 ± 3.0	11.3 ± 3.0	10.9 ± 3.0	<0.001
WC, cm	64.9 ± 10.3	68.9 ± 11.8	65.4 ± 10.5	66.0 ± 10.4	62.9 ± 9.2	<0.001
BMI, kg/m^2^	18.5 ± 3.6	20.1 ± 4.2	18.7 ± 3.7	19.0 ± 3.7	17.7 ± 3.2	<0.001
***Residence area (n, %)***						<0.001
Urban	24,519 (61.0)	2964 (57.2)	9280 (64.3)	2164 (53.3)	10,111 (61.2)	
Rural	15,678 (39.0)	2218 (42.8)	5156 (35.7)	1895 (46.7)	6409 (38.8)	
***Sex (n, %)***						<0.001
Boys	21,197 (52.7)	2848 (55.0)	7710 (53.4)	2074 (51.1)	8565 (51.8)	
Girls	19,000 (47.3)	2334 (45.0)	6726 (46.6)	1985 (48.9)	7955 (48.2)	
***Single child (n, %)***						<0.001
Yes	26,526 (66.0)	3029 (58.5)	9916 (68.7)	2348 (57.8)	11,233 (68.0)	
No	13,671 (34.0)	2153 (41.5)	4520 (31.3)	1711 (42.2)	5287 (32.0)	
***Breastfeeding (n, %)***						
Yes	34,256 (85.2)	4430 (85.5)	12,302 (85.2)	3463 (85.3)	14,061 (85.1)	0.925
No	5941 (14.8)	752 (14.5)	2134 (14.8)	596 (14.7)	2459 (14.9)	
***Types of obesity (n, %)***						
Normal	29,340 (73.0)	2979 (57.5)	9978 (69.1)	2916 (71.8)	13,467 (81.5)	<0.001
General obesity	350 (0.9)	55 (1.1)	145 (1.0)	39 (1.0)	111 (0.7)	
Simple abdominal obesity	966 (2.4)	126 (2.4)	393 (2.7)	91 (2.2)	356 (2.2)	
Compound obesity	9541 (23.7)	2022 (39.0)	3920 (27.2)	1013 (25.0)	2586 (15.7)	
***Dietary behaviors (n, %)***						
Meat consumption ≤ 3 services/week	10,399 (25.9)	1540 (29.7)	3680 (25.5)	1178 (29.0)	4001 (24.2)	<0.001
SSBs consumption ≤ 3 sevices/week	31,070 (77.3)	3864 (74.6)	11,243 (77.9)	3071 (75.7)	12,892 (78.0)	<0.001
Eating breakfast ≥ 6 days/week	35,537 (88.4)	4496 (86.8)	12,855 (89.0)	3506 (86.4)	14,680 (88.9)	<0.001
Fast-food consumption < 1 times/month	21,316 (53.0)	2908 (56.1)	7458 (51.7)	2324 (57.3)	8626 (52.2)	<0.001
***Physical activity (n, %)***						
MVPA ≥ 1 h/day	19,526 (48.6)	2633(50.8)	6869(47.6)	2040(50.3)	7984(48.3)	<0.001
***Parental factors (n, %)***						
Paternal BMI	24.1 ± 3.2	27.0 ± 2.7	26.6 ± 2.2	21.8 ± 1.6	21.6 ± 1.7	<0.001
Maternal BMI	22.2 ± 3.0	26.5 ± 2.7	21.1 ± 1.7	26.3 ± 2.4	20.8 ± 1.8	<0.001
***Paternal highest educational attainment (n, %)***						
Primary school or below	2867 (7.1)	492 (9.5)	797 (5.5)	469 (11.6)	1109 (6.7)	<0.001
Junior high school and Senior high school	25,959 (64.6)	3542 (68.4)	8913 (61.7)	2875 (70.8)	10,629 (64.3)	
Junior college or above	11,371 (28.3)	1148 (22.2)	4726 (32.7)	715 (17.6)	4782 (28.9)	
***Maternal highest educational attainment (n, %)***						
Primary school or below	4016 (10.0)	683 (13.2)	1168 (8.1)	623 (15.3)	1542 (9.3)	<0.001
Junior high school and Senior high school	25,835 (64.3)	3515 (67.8)	8964 (62.1)	2774 (68.3)	10,582 (64.1)	
Junior college or above	10,346 (25.7)	984 (19.0)	4304 (29.8)	662 (16.3)	4396 (26.6)	
***Dietary behaviors (n, %)***						
Meat consumption ≤ 3 services/week	10,281 (25.6)	1637 (31.6)	3683 (25.5)	1202 (29.6)	3759 (22.8)	<0.001
SSBs consumption ≤ 3 sevices/week	35,586 (88.5)	4552 (87.8)	12,809 (88.7)	3593 (88.5)	14,632 (88.6)	0.391
Fast-food consumption < 1 times/month	26,808 (66.7)	3594 (69.4)	9406 (65.2)	2916 (71.8)	10,892 (65.9)	<0.001
Eating breakfast ≥ 6 days/week	35,627 (88.6)	4556 (87.9)	12,772 (88.5)	3583 (88.3)	14,716 (89.1)	0.079
***Physical activity (n, %)***						
MVPA ≥ 1 h/day	19,526 (48.6)	2633 (50.8)	6869 (47.6)	2040 (50.3)	7984 (48.3)	<0.001

Notes: BMI, body mass index; WC, waist circumference; SSBs, Sugar-sweetened beverages; MVPA, moderate to vigorous physical activity.

**Table 2:** Association of OW/OB between parents and offspring.

Variables	General Obesity OR (95% CI)	Simple Abdominal Obesity OR (95% CI)	Compound Obesity OR (95% CI)	High WC Level OR (95% CI)	High BMI Level OR (95% CI)
Both parental OW/OB	2.66 (1.91–3.71) ***	1.72 (1.40–2.12) ***	4.04 (3.75–4.35) ***	3.65 (3.38–3.93) ***	3.92 (3.65–4.21) ***
Only paternal OW/OB	1.80 (1.41–2.32) ***	1.46 (1.26–1.69) ***	2.06 (1.94–2.18) ***	1.99 (1.87–2.11) ***	2.03 (1.91–2.14) ***
Only maternal OW/OB	1.98 (1.37–2.87) **	1.28 (1.01–1.62) *	2.11 (1.93–2.30) ***	1.96 (1.79–2.15) ***	2.09 (1.92–2.27) ***
No parental OW/OB	1 (Reference)	1 (Reference)	1 (Reference)	1 (Reference)	1 (Reference)

Notes: Adjust for offspring age, sex, resident area, single-child status, breastfeeding, MVPA status; parental highest educational attainment, and MVPA status; * *p* < 0.05, ** *p* < 0.01, *** *p* < 0.001.

**Table 3:** Association of OW/OB between parents and offspring by the combination of dietary behaviors in two generations.

Variables	General Obesity OR (95% CI)	Simple Abdominal Obesity OR (95% CI)	Compound Obesity OR (95% CI)	High WC Level OR (95% CI)	High BMI Level OR (95% CI)
***Both parents and offspring had unhealthy dietary behaviors***					
Both parental OW/OB	2.80 (1.87–4.18) ***	1.98 (1.56–2.52) ***	4.05 (3.72–4.41) ***	3.67 (3.36–4.00) ***	3.93 (3.60–4.27) ***
Only paternal OW/OB	1.82 (1.35–2.44) ***	1.58 (1.34–1.88) ***	2.09 (1.96–2.23) ***	2.04 (1.90–2.18) ***	2.05 (1.92–2.19) ***
Only maternal OW/OB	1.98 (1.25–3.12) **	1.40 (1.06–1.84) *	2.12 (1.92–2.35) ***	1.98 (1.78–2.20) ***	2.10 (1.90–2.32) ***
No parental OW/OB	1 (Reference)	1 (Reference)	1 (Reference)	1 (Reference)	1 (Reference)
***Only parents had unhealthy dietary behaviors***					
Both parental OW/OB	2.33 (0.83–6.54)	1.10 (0.55–2.19)	3.81 (2.93–4.96) ***	3.26 (2.49–4.27) ***	3.71 (2.87–4.81) ***
Only paternal OW/OB	1.09 (0.44–2.71)	0.93 (0.58–1.51)	1.85 (1.50–2.30) ***	1.68 (1.35–2.10) ***	1.82 (1.47–2.24) ***
Only maternal OW/OB	2.90 (1.07–7.84) *	0.52 (0.20–1.34)	1.84 (1.35–2.52) ***	1.51 (1.08–2.11) ***	1.94 (1.43–2.62) ***
No parental OW/OB	1 (Reference)	1 (Reference)	1 (Reference)	1 (Reference)	1 (Reference)
***Only offspring had unhealthy dietary behaviors***					
Both parental OW/OB	2.20 (0.92–5.30)	0.92 (0.47–1.83)	4.03 (3.27–4.97) ***	3.52 (2.83–4.37) ***	3.96 (3.22–4.87) ***
Only paternal OW/OB	1.85 (0.92–3.73)	1.13 (0.73–1.75)	1.94 (1.62–2.31) ***	1.79 (1.49–2.16) ***	1.93 (1.62–2.29) ***
Only maternal OW/OB	1.20 (0.38–3.75)	1.18 (0.62–2.24)	2.16 (1.70–2.75) ***	2.10 (1.63–2.69) ***	2.11 (1.66–2.67) ***
No parental OW/OB	1 (Reference)	1 (Reference)	1 (Reference)	1 (Reference)	1 (Reference)
***Both parents and offspring had healthy dietary behaviors***					
Both parental OW/OB	1.75 (0.48–6.42)	2.27 (0.82–6.26)	3.62 (2.61–5.01) ***	3.58 (2.55–5.02) ***	3.45 (2.51–4.74) ***
Only paternal OW/OB	2.28 (0.82–6.31)	1.84 (0.77–4.38)	1.98 (1.49–2.64) ***	2.00 (1.47–2.72) ***	1.97 (1.49–2.61) ***
Only maternal OW/OB	1.45 (0.35–6.00)	1.94 (0.67–5.61)	1.75 (1.20–2.56) **	1.78 (1.20–2.66) *	1.71 (1.18–2.48) **
No parental OW/OB	1 (Reference)	1 (Reference)	1 (Reference)	1 (Reference)	1 (Reference)

* *p* < 0.05, ** *p* < 0.01, *** *p* < 0.001. Adjust for offspring age, sex, resident area, single-child status, breastfeeding, MVPA status; parental highest educational attainment, and MVPA status.

## Data Availability

The raw data supporting the conclusions of this article will be made available by the authors, without undue reservation.

## Supplementary Materials

The following supporting information can be downloaded at: https://www.mdpi.com/article/10.3390/nu14214625/s1, Figure S1: Correlation coefficients between parental and offspring dietary behaviors and factors. Table S1: Information on covariates in the questionnaire. Table S2: Sex-age BMI screening for overweight and obesity in school-age children and adolescents aged 6 to 18 years in China. Table S3: P75 and P90 waist circumference of children and adolescents aged 7-18 years in China. Table S4: Definition of obesity status. Table S5: Descriptive characteristics of the study population analysis by Bonferroni correction. Table S6: Association of OW/OB between parents and offspring, stratified by parental unhealthy/healthy dietary behaviors. Table S7: Association of OW/OB between parents and offspring, stratified by offspring unhealthy/healthy dietary behaviors. Table S8: Number of each missing variable. Table S9: Sensitive analysis in the association of OW/OB between parents and offspring. Table S10: Association of OW/OB between parents and offspring in sensitive analysis, stratified by parental unhealthy/healthy dietary behaviors. Table S11: Sensitive analysis in the association of OW/OB between parents and offspring, stratified by offspring unhealthy/healthy dietary behaviors. Table S12: Association of OW/OB between parents and offspring in sensitive analysis by to the combination of dietary behaviors in two generations.
