# Supplementary material for: Influence of Parental and Offspring Dietary Behaviors on the Association of Overweight and Obesity between Two Generations: Results from a Cross-Sectional Analysis of Parent-Offspring Trios in China
Source: Nutrients. 2022 Nov 2;14(21):4625. doi: 10.3390/nu14214625 (PMC9658054; doi:10.3390/nu14214625)
Supplement: Supplementary file 1 [file nutrients-14-04625-s001.zip › nutrients-1981634-supplementary.pdf]

Supplementary Material

1. Supplementary Figure

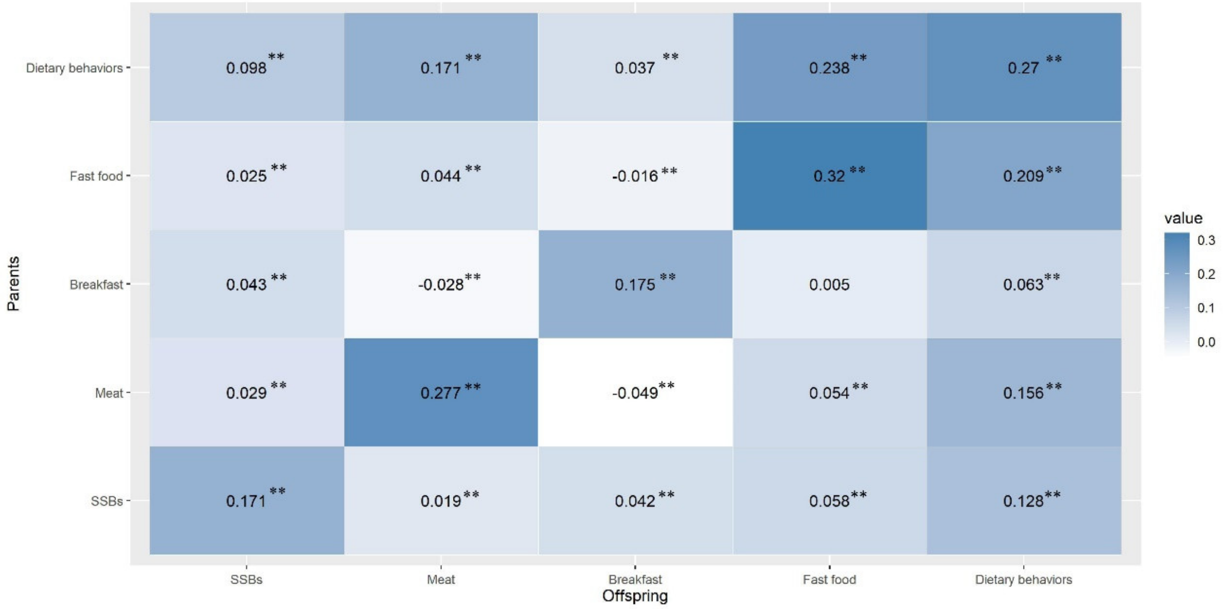

Supplementary Figure S1. Correlation coefficients between parental and offspring dietary behaviors and factors. SSBs, Sugar-sweetened beverages; ** p <0.01.

2. Supplementary Table

Supplementary Table S1. Information on covariates in the questionnaire

Covariate	Question	Answer
Residence area	"Where your family is located?"	1.Rural 2.Urban
Single-child status	"Is your child a single child?"	1.Yes 2.No
Breastfeeding	"Is your child breastfeeding?"	1.Yes 2. No
Paternal educational attainment	"What is the father's educational attainment?"	1.Not been to school<br>2. Primary school<br>3. Junior high school<br>4. Senior high school<br>5. Junior college<br>6. Undergraduate college
Maternal educational attainment	"What is the mother's educational attainment?"	1.Not been to school<br>2. Primary school<br>3. Junior high school<br>4. Senior high school<br>5. Junior college<br>6. Undergraduate college
MVPA	"How many days, during the past 7 days before the survey, have you had moderate/vigorous physical activity? How long in one day ?"	_____days_____hours__ minutes
SSBs	"How many days, during the past 7 days before the survey, have you drunk SSBs (such as Coca-Cola, Sprite, orange juice, Nutrition Express, Red Bull, and all other sorts of sodas and sugary juices)? How many servings in	_____days_____servings

	one day?” (One serving approximately 250ml)	
Meat	“How many days, during the past 7 days before the survey, have you eaten meat? How many portions in one day?” (One portion of meat equaled the size of an adult’s palm)	_____days_____portions
Fast foods	“How many times, during the past 7 days before the survey, have you eaten fried foods?”	_____times
Breakfast	“How many days, during the past 7 days before the survey, have you eaten breakfast ?”	_____days

Note: The questions about dietary intake are same for parents and offspring.

Supplementary Table S2. Sex-age BMI screening for overweight and obesity in school-age children and adolescents aged 6 to 18 years in China.

Age	Boys		Girls	
(Year)	Overweight	Obesity	Overweight	Obesity
6.0~	16.4	17.7	16.2	17.5
6.5~	16.7	18.1	16.5	18.0
7.0~	17.0	18.7	16.8	18.5
7.5~	17.4	19.2	17.2	19.0
8.0~	17.8	19.7	17.6	19.4
8.5~	18.1	20.3	18.1	19.9
9.0~	18.5	20.8	18.5	20.4
9.5~	18.9	21.4	19.0	21.0
10.0~	19.2	21.9	19.5	21.5
10.5~	19.6	22.5	20.0	22.1
11.0~	19.9	23.0	20.5	22.7
11.5~	20.3	23.6	21.1	23.3
12.0~	20.7	24.1	21.5	23.9
12.5~	21.0	24.7	21.9	24.5
13.0~	21.4	25.2	22.2	25.0
13.5~	21.9	25.7	22.6	25.6
14.0~	22.3	26.1	22.8	25.9
14.5~	22.6	26.4	23.0	26.3
15.0~	22.9	26.6	23.2	26.6
15.5~	23.1	26.9	23.4	26.9
16.0~	23.3	27.1	23.6	27.1
16.5~	23.5	27.4	23.7	27.4
17.0~	23.7	27.6	23.8	27.6
17.5~	23.8	27.8	23.9	27.8
18.0~	24.0	28.0	24.0	28.0

Supplementary Table S3. P_{75} and P_{90} waist circumference of children and adolescents aged 7-18 years in China.

Age (Year)	Boys		Girls	
	**P_{75}**	**P_{90}**	**P_{75}**	**P_{90}**
7	58.4	63.6	55.8	60.2
8	60.8	66.8	57.6	62.5
9	63.4	70.0	59.8	65.1
10	65.9	73.1	62.2	67.8
11	68.1	75.6	64.6	70.4
12	69.8	77.4	66.8	72.6
13	71.3	78.6	68.5	74.0
14	72.6	79.6	69.6	74.9
15	73.8	80.5	70.4	75.5
16	74.8	81.3	70.9	75.8
17	75.7	82.1	71.2	76.0
18	76.8	83.0	71.3	76.1

Supplementary Table S4. Definition of obesity status.

	**Normal**	**General Obesity**	**Simple Abdominal Obesity**	**Compound Obesity**
BMI	< the sex-age's "overweight" threshold	⩾ the sex-age's "overweight" threshold	< the sex-age's "overweight" threshold	⩾ the sex-age's "overweight" threshold
WC	< P_{90} waist circumference of children and adolescents	< P_{90} waist circumference of children and adolescents	⩾ P_{90} waist circumference of children and adolescents	⩾ P_{90} waist circumference of children and adolescents

Supplementary Table S5. Descriptive characteristics of the study population analysis by Bonferroni correction

Characteristics	Total n = 40197	Parental OW/OB Status			
		Both Parental OW/OB n = 5182	Only Paternal OW/OB n = 14366	Only Maternal OW/OB n = 4034	None OW/OB n = 16425
Offspring factors					
Age, year	10.9±3.0	11.0±3.0 a	10.7±3.0 b	11.3±3.0 c	10.9±3.0 a
WC, cm	64.9±10.3	68.9±11.8 a	65.4±10.5 b	66.0±10.4 b	62.9±9.2 c
BMI, kg/m^2	18.5±3.6	20.1±4.2 a	18.7±3.7 b	19.0±3.7 c	17.7±3.2 d
Residence area (n, %)					
Urban	24519 (61.0)	2964 (57.2) a	9280 (64.3) b	2164 (53.3) c	10111 (61.2) d
Rural	15678 (39.0)	2218 (42.8) a	5156 (35.7) b	1895 (46.7) c	6409 (38.8) d
Sex (n, %)					
Boys	21197 (52.7)	2848 (55.0) a	7710 (53.4) ab	2074 (51.1) bc	8565 (51.8) c
Girls	19000 (47.3)	2334 (45.0) a	6726 (46.6) ab	1985 (48.9) bc	7955 (48.2) c
Single child (n, %)					
Yes	26526 (66.0)	3029 (58.5) a	9916 (68.7) b	2348 (57.8) a	11233 (68.0) b
No	13671 (34.0)	2153 (41.5) a	4520 (31.3) b	1711 (42.2) a	5287 (32.0) b
Breastfeeding (n, %)					
Yes	34256 (85.2)	4430 (85.5) a	12302 (85.2) a	3463 (85.3) a	14061 (85.1) a
No	5941 (14.8)	752 (14.5) a	2134 (14.8) a	596 (14.7) a	2459 (14.9) a
Types of obesity (n, %)					
Normal	29340 (73.0)	2979 (57.5) a	9978 (69.1) b	2916 (71.8) c	13467 (81.5) d
General obesity	350 (0.9)	55 (1.1) a	145 (1.0) a	39 (1.0) ab	111 (0.7) b
Simple abdominal obesity	966 (2.4)	126 (2.4) ab	393 (2.7) b	91 (2.2) ab	356 (2.2) a

Compound obesity	9541 (23.7)	2022 (39.0) a	3920 (27.2) b	1013 (25.0) c	2586 (15.7) d
Dietary behaviors (n, %)					
meat consumption≤3 services/week	10399 (25.9)	1540 (29.7) a	3680 (25.5) b	1178 (29.0) a	4001 (24.2) b
SSBs consumption≤3sevices/week	31070 (77.3)	3864 (74.6) a	11243 (77.9) b	3071 (75.7) a	12892 (78.0) b
Eating breakfast≥6 days/week	35537 (88.4)	4496 (86.8) a	12855 (89.0) b	3506 (86.4) a	14680 (88.9) b
Fast food consumption<1 times/month	21316 (53.0)	2908 (56.1) a	7458 (51.7) b	2324 (57.3) a	8626 (52.2) b
Physical activity (n, %)					
MVPA≥1 h/day	19526(48.6)	2633(50.8) a	6869(47.6) b	2040(50.3) ac	7984(48.3) bc
Parental factors (n, %)					
Paternal BMI	24.1±3.2	27.0±2.7 a	26.6±2.2 b	21.8±1.6 c	21.6±1.7 d
Maternal BMI	22.2±3.0	26.5±2.7 a	21.1±1.7 b	26.3±2.4 c	20.8±1.8 d
Paternal highest educational attainment (n, %)					
Primary school or below	2867 (7.1)	492 (9.5) a	797 (5.5) b	469 (11.6) c	1109 (6.7) d
Junior high school and Senior high school	25959 (64.6)	3542 (68.4) a	8913 (61.7) b	2875 (70.8) a	10629 (64.3) c
Junior college or above	11371 (28.3)	1148 (22.2) a	4726 (32.7) b	715 (17.6) c	4782 (28.9) d
Maternal highest educational attainment (n, %)					
Primary school or below	4016 (10.0)	683 (13.2) a	1168 (8.1) b	623 (15.3) c	1542 (9.3) d
Junior high school and Senior high school	25835 (64.3)	3515 (67.8) a	8964 (62.1) b	2774 (68.3) a	10582 (64.1) c
Junior college or above	10346 (25.7)	984 (19.0) a	4304 (29.8) b	662 (16.3) c	4396 (26.6) d
Dietary behaviors (n, %)					
meat consumption≤3 services/week	10281 (25.6)	1637 (31.6) a	3683 (25.5) b	1202 (29.6) a	3759 (22.8) c
SSBs consumption≤3sevices/week	35586 (88.5)	4552 (87.8) a	12809 (88.7) a	3593 (88.5) a	14632 (88.6) a
Fast food consumption<1 times/month	26808 (66.7)	3594 (69.4) a	9406 (65.2) b	2916 (71.8) a	10892 (65.9) b
Eating breakfast≥6days/week	35627 (88.6)	4556 (87.9) a	12772 (88.5) a	3583 (88.3) a	14716 (89.1) a
Physical activity (n, %)					
MVPA≥1 h/day	19526(48.6)	2633(50.8) a	6869(47.6) b	2040(50.3) c	7984(48.3) b

Bonferroni correction was used to examine the diversity between different groups. There is significant difference between two groups without the same alphabets in the lower right corner, while there is non-significant difference between two groups if exist same alphabets. A two-sided p-value < 0.0083 was considered statistically significant. BMI, body mass index; WC, waist circumference; SSBs, Sugar-sweetened beverages; MVPA, moderate to vigorous physical activity.

Supplementary Table S6. Association of OW/OB between parents and offspring, stratified by parental unhealthy/healthy dietary behaviors.

Variables	General obesity OR (95%CI)	Simple abdominal obesity OR (95%CI)	Compound obesity OR (95%CI)	High WC level OR (95%CI)	High BMI level OR (95%CI)
Parents had unhealthy dietary behaviors					
Both parental OW/OB	2.73 (1.88-3.97) ***	1.83 (1.46-2.30) ***	4.00 (3.69-4.34) ***	3.60 (3.31-3.91) ***	3.88 (3.58-4.20) ***
Only paternal OW/OB	1.73 (1.31-2.28) ***	1.49 (1.27-1.75) ***	2.07 (1.94-2.20) ***	2.00 (1.87-2.13) ***	2.03 (1.91-2.16) ***
Only maternal OW/OB	2.14 (1.42-3.22) ***	1.26 (0.97-1.65)	2.09 (1.90-2.30) ***	1.92 (1.74-2.13) ***	2.07 (1.89-2.28) ***
No parental OW/OB	1 (Reference)	1 (Reference)	1 (Reference)	1 (Reference)	1 (Reference)
Parents had healthy dietary behaviors					
Both parental OW/OB	2.11 (1.02-4.35) *	1.17 (0.67-2.03)	3.90 (3.27-4.65) ***	3.53 (2.94-4.23) ***	3.79 (3.19-4.51) ***
Only paternal OW/OB	1.96 (1.10-3.48) *	1.26 (0.85-1.86)	2.96 (1.68-2.27) ***	1.86 (1.58-2.18) ***	1.94 (1.68-2.25) ***
Only maternal OW/OB	1.29 (0.53-3.11)	1.30 (0.76-2.23)	2.04 (1.67-2.51) ***	2.00 (1.62-2.48) ***	1.99 (1.63-2.43) ***
No parental OW/OB	1 (Reference)	1 (Reference)	1 (Reference)	1 (Reference)	1 (Reference)

*P<0.05, **P<0.01, ***P<0.001

Adjust for offspring age, sex, resident area, single-child status, breastfeeding, MVPA status, SSBs consumption, meat consumption, breakfast eating, fast food consumption; parental highest educational attainment, and MVPA status.

Supplementary Table S7. Association of OW/OB between parents and offspring, stratified by offspring unhealthy/healthy dietary behaviors.

Variables	General Obesity OR (95%CI)	Simple Abdominal Obesity OR (95%CI)	Compound Obesity OR (95%CI)	High WC Level OR (95%CI)	High BMI Level OR (95%CI)
Offspring had unhealthy dietary behaviors					
Both parental OW/OB	2.68 (1.86-3.86) ***	1.78 (1.42-2.24) ***	4.03 (3.73-4.37) ***	3.63 (3.34-3.94) ***	3.92 (3.62-4.24) ***
Only paternal OW/OB	1.83 (1.39-2.39) ***	1.51 (1.29-1.77) ***	2.07 (1.94-2.20) ***	2.00 (1.87-2.13) ***	2.03 (1.91-2.16) ***
Only maternal OW/OB	1.80 (1.18-2.75) **	1.36 (1.06-1.76) *	2.13 (1.94-2.33) ***	2.00 (1.81-2.20) ***	2.10 (1.91-2.30) ***
No parental OW/OB	1 (Reference)	1 (Reference)	1 (Reference)	1 (Reference)	1 (Reference)
Offspring had healthy dietary behaviors					
Both parental OW/OB	1.98 (0.88-4.46)	1.34 (0.77-2.34)	3.70 (3.01-4.53) ***	3.37 (2.74-4.16) ***	3.56 (2.91-4.35) ***
Only paternal OW/OB	1.47 (0.75-2.86)	1.09 (0.72-1.66)	1.91 (1.61-2.27) ***	1.80 (1.50-2.15) ***	1.88 (1.59-2.23) ***
Only maternal OW/OB	2.19 (0.97-4.94)	0.84 (0.43-1.64)	1.83 (1.44-2.32) ***	1.63 (1.27-2.11) ***	1.86 (1.47-2.35) ***
No parental OW/OB	1 (Reference)	1 (Reference)	1 (Reference)	1 (Reference)	1 (Reference)

*P<0.05, **P<0.01, ***P<0.001

Adjust for offspring age, sex, resident area, single-child status, breastfeeding, MVPA status; parental highest educational attainment, MVPA status, SSBs consumption, meat consumption, breakfast eating, and fast food consumption.

Supplementary Table S8. Number of each missing variable.

Variables	Missing Records n (%)	Effective Records n (%)
Offspring variables		
Breastfeeding	481 (1.2%)	39716 (98.8%)
MVPA	7358 (18.3%)	32839 (81.7%)
SSBs consumption/week	1045 (2.6%)	39152 (97.4%)
Meat consumption/week	444 (1.1%)	39753 (98.9%)
Frequency of eating fast food	427 (1.1%)	39770 (98.9 %)
Frequency of eating breakfast	36 (0.1%)	40161 (99.9%)
Parental variables		
MVPA	3098 (7.7%)	37099 (92.3%)
SSBs consumption/week	1083 (2.7%)	39114 (97.3%)
Meat consumption/week	617 (1.5%)	39580 (98.5%)
Frequency of eating fast food	332 (0.8%)	39865 (99.2%)
Frequency of eating breakfast	33 (0.1%)	40164 (99.9%)

SSBs: sugar-sweetened beverages; MVPA: moderate to vigorous physical activity.

Supplementary Table S9. Sensitive analysis in the association of OW/OB between parents and offspring.

Variables	General obesity OR (95%CI)	Simple abdominal obesity OR (95%CI)	Compound obesity OR (95%CI)	High WC level OR (95%CI)	High BMI level OR (95%CI)
Both parental OW/OB	2.44 (1.62-3.67) ***	1.67 (1.30-2.14) ***	3.81 (3.49-4.15) ***	3.46 (3.16-3.78) ***	3.70 (3.39-4.03) ***
Only paternal OW/OB	1.71 (1.26-2.31) **	1.46 (1.23-1.73) ***	1.99 (1.86-2.13) ***	1.93 (1.80-2.07) ***	1.96 (1.83-2.09) ***
Only maternal OW/OB	1.67 (1.03-2.71) *	1.26 (0.95-1.66)	2.03 (1.83-2.25) ***	1.98 (1.78-2.21) ***	2.00 (1.81-2.22) ***
No parental OW/OB	1 (Reference)	1 (Reference)	1 (Reference)	1 (Reference)	1 (Reference)

*P<0.05, **P<0.01, ***P<0.001

Adjust for offspring factors including age, sex, resident area, single-child status, breastfeeding, MVPA status; parental factors including highest educational attainment, and MVPA.

Supplementary Table S10. Association of OW/OB between parents and offspring in sensitive analysis, stratified by parental unhealthy/healthy dietary behaviors.

Variables	General obesity OR (95%CI)	Simple abdominal obesity OR (95%CI)	Compound obesity OR (95%CI)	High WC level OR (95%CI)	High BMI level OR (95%CI)
Parents had unhealthy dietary behaviors					
Both parental OW/OB	2.39 (1.51-3.77) ***	1.74 (1.33-2.28) ***	3.79 (3.44-4.17) ***	3.44 (3.11-3.80) ***	3.67 (3.33-4.04) ***
Only paternal OW/OB	1.44 (1.03-2.02) *	1.48 (1.23-1.79) ***	2.01 (1.86-2.16) ***	1.95 (1.80-2.11) ***	1.96 (1.82-2.11) ***
Only maternal OW/OB	1.78 (1.06-2.99) *	1.24 (0.91-1.70)	2.00 (1.78-2.24) ***	1.93 (1.71-2.17) ***	1.98 (1.77-2.21) ***
No parental OW/OB	1 (Reference)	1 (Reference)	1 (Reference)	1 (Reference)	1 (Reference)
Parents had healthy dietary behaviors					
Both parental OW/OB	2.75 (1.04-7.28) *	1.22 (0.64-2.30)	3.58 (2.90-4.41) ***	3.24 (2.60-4.02) ***	3.53 (2.87-4.34) ***
Only paternal OW/OB	3.28 (1.52-7.08) **	1.29 (0.81-2.05)	1.84 (1.54-2.20) ***	1.77 (1.46-2.14) ***	1.87 (1.57-2.23) ***
Only maternal OW/OB	1.25 (0.33-4.70)	1.27 (0.66-2.43)	2.03 (1.60-2.59) ***	2.09 (1.62-2.69) ***	2.00 (1.57-2.54) ***
No parental OW/OB	1 (Reference)	1 (Reference)	1 (Reference)	1 (Reference)	1 (Reference)

*P<0.05, **P<0.01, ***P<0.001

Adjust for offspring age, sex, resident area, single-child status, breastfeeding, MVPA status, SSBs consumption, meat consumption, breakfast eating, fast food consumption; parental highest educational attainment, and MVPA status.

Supplementary Table S11. Sensitive analysis in the association of OW/OB between parents and offspring, stratified by offspring unhealthy/healthy dietary behaviors.

Variables	General obesity OR (95%CI)	Simple abdominal obesity OR (95%CI)	Compound obesity OR (95%CI)	High WC level OR (95%CI)	High BMI level OR (95%CI)
Offspring had unhealthy dietary behaviors					
Both parental OW/OB	2.40 (1.52-3.77) ***	1.75 (1.34-2.29) ***	3.77 (3.43-4.14) ***	3.48 (3.16-3.84) ***	3.66 (3.33-4.01) ***
Only paternal OW/OB	1.65 (1.18-2.29) **	1.50 (1.25-1.81) ***	2.00 (1.86-2.14) ***	1.95 (1.81-2.11) ***	1.96 (1.82-2.10) ***
Only maternal OW/OB	1.47 (0.85-2.57)	1.32 (0.98-1.79)	2.02 (1.81-2.26) ***	2.01 (1.79-2.25) ***	1.98 (1.78-2.21) ***
No parental OW/OB	1 (Reference)	1 (Reference)	1 (Reference)	1 (Reference)	1 (Reference)
Offspring had healthy dietary behaviors					
Both parental OW/OB	2.05 (0.77-5.45)	1.21 (0.61-2.39)	3.65 (2.86-4.67) ***	2.92 (2.27-3.75) ***	3.53 (2.78-4.50) ***
Only paternal OW/OB	1.79 (0.82-3.92)	1.17 (0.72-1.91)	1.86 (1.52-2.29) ***	1.70 (1.37-2.10) ***	1.85 (1.51-2.26) ***
Only maternal OW/OB	2.09 (0.75-5.81)	0.99 (0.47-2.11)	1.92 (1.44-2.55) ***	1.70 (1.26-2.29) ***	1.92 (1.45-2.54) ***
No parental OW/OB	1 (Reference)	1 (Reference)	1 (Reference)	1 (Reference)	1 (Reference)

*P<0.05, **P<0.01, ***P<0.001

Adjust for offspring age, sex, resident area, single-child status, breastfeeding, MVPA status; parental highest educational attainment, MVPA status, SSBs consumption, meat consumption, breakfast eating, and fast food consumption.

Supplementary Table S12. Association of OW/OB between parents and offspring in sensitive analysis by to the combination of dietary behaviors in two generations.

**Variables**	**General obesity OR (95%CI)**	**Simple abdominal obesity OR (95%CI)**	**Compound obesity OR (95%CI)**	**High WC level OR (95%CI)**	**High BMI level OR (95%CI)**
Both parents and offspring had unhealthy dietary behaviors					
Both parental OW/OB	2.27 (1.38-3.75) **	1.91 (1.43-2.54) ***	3.80 (3.43-4.21) ***	3.54 (3.18-3.93) ***	3.67 (3.32-4.06) ***
Only paternal OW/OB	1.51 (1.06-2.15) *	1.58 (1.30-1.92) ***	2.02 (1.87-2.19) ***	1.99 (1.84-2.16) ***	1.98 (1.83-2.13) ***
Only maternal OW/OB	1.64 (0.92-2.91)	1.35 (0.97-1.87)	2.02 (1.79-2.28) ***	1.97 (1.74-2.24) ***	1.99 (1.77-2.24) ***
No parental OW/OB	1 (Reference)	1 (Reference)	1 (Reference)	1 (Reference)	1 (Reference)
Only parents had unhealthy dietary behaviors					
Both parental OW/OB	2.79 (0.86-9.12)	0.96 (0.41-2.26)	3.98 (2.89-5.48) ***	2.83 (1.05-3.90) ***	3.92 (2.86-5.36) ***
Only paternal OW/OB	0.86 (0.27-2.76)	0.93 (0.53-1.64)	1.86 (1.43-2.40) ***	1.59 (1.23-2.07) ***	1.80 (1.40-2.32) ***
Only maternal OW/OB	2.73 (0.77-9.71)	0.72 (0.27-1.91)	1.87 (1.28-2.73) **	1.62 (1.10-2.40) *	1.93 (1.33-2.79) ***
No parental OW/OB	1 (Reference)	1 (Reference)	1 (Reference)	1 (Reference)	1 (Reference)
Only offspring had unhealthy dietary behaviors					
Both parental OW/OB	4.25 (1.26-14.29) *	1.01 (0.47-2.17)	3.72 (2.89-4.78) ***	3.32 (2.56-4.31) ***	3.73 (2.91-4.78) ***
Only paternal OW/OB	3.30 (1.18-9.21) *	1.07 (0.64-1.80)	1.84 (1.49-2.27) ***	1.72 (1.38-2.15) ***	1.87 (1.52-2.30) ***
Only maternal OW/OB	0.92 (0.11-8.03)	1.15 (0.53-2.47)	2.04 (1.53-2.73) ***	2.21 (1.64-2.98) ***	2.01 (1.51-2.68) ***
No parental OW/OB	1 (Reference)	1 (Reference)	1 (Reference)	1 (Reference)	1 (Reference)
Both parents and offspring had healthy dietary behaviors					
Both parental OW/OB	1.28 (0.22-7.26)	2.48 (0.69-8.83)	3.29 (2.24-4.84) ***	3.11(2.08-4.65) ***	3.13 (2.14-4.57) ***
Only paternal OW/OB	3.38 (1.04-10.98)	2.64 (0.88-7.91)	1.82 (1.29-2.57) ***	1.87 (1.30-2.69) **	1.85 (1.33-2.59) ***

Only maternal OW/OB	1.45 (0.25-8.26)	2.08 (0.54-8.02)	1.91 (1.23-2.97) **	1.80 (1.12-2.288) *	1.86 (1.21-2.87) **
No parental OW/OB	1 (Reference)	1 (Reference)	1 (Reference)	1 (Reference)	1 (Reference)

*P<0.05, **P<0.01, ***P<0.001

Adjust for offspring age, sex, resident area, single-child status, breastfeeding, MVPA status; parental highest educational attainment, and MVPA status.
